# Fish and chips: Various methodologies demonstrate utility of a 16,006-gene salmonid microarray

**DOI:** 10.1186/1471-2164-6-126

**Published:** 2005-09-15

**Authors:** Kristian R von Schalburg, Matthew L Rise, Glenn A Cooper, Gordon D Brown, A Ross Gibbs, Colleen C Nelson, William S Davidson, Ben F Koop

**Affiliations:** 1 Centre for Biomedical Research, University of Victoria, Victoria, British Columbia, V8W 3N5, Canada; 2 Great Lakes WATER Institute, University of Wisconsin-Milwaukee, Milwaukee, WI, 53204, USA; 3 The Prostate Centre at Vancouver General Hospital, Gene Array Facility, Vancouver, British Columbia, V6H 3Z6, Canada; 4 Department of Molecular Biology and Biochemistry, Simon Fraser University, Burnaby, British Columbia, V5A 1S6, Canada

## Abstract

**Background:**

We have developed and fabricated a salmonid microarray containing cDNAs representing 16,006 genes. The genes spotted on the array have been stringently selected from Atlantic salmon and rainbow trout expressed sequence tag (EST) databases. The EST databases presently contain over 300,000 sequences from over 175 salmonid cDNA libraries derived from a wide variety of tissues and different developmental stages. In order to evaluate the utility of the microarray, a number of hybridization techniques and screening methods have been developed and tested.

**Results:**

We have analyzed and evaluated the utility of a microarray containing 16,006 (16K) salmonid cDNAs in a variety of potential experimental settings. We quantified the amount of transcriptome binding that occurred in cross-species, organ complexity and intraspecific variation hybridization studies. We also developed a methodology to rapidly identify and confirm the contents of a bacterial artificial chromosome (BAC) library containing Atlantic salmon genomic DNA.

**Conclusion:**

We validate and demonstrate the usefulness of the 16K microarray over a wide range of teleosts, even for transcriptome targets from species distantly related to salmonids. We show the potential of the use of the microarray in a variety of experimental settings through hybridization studies that examine the binding of targets derived from different organs and tissues. Intraspecific variation in transcriptome expression is evaluated and discussed. Finally, BAC hybridizations are demonstrated as a rapid and accurate means to identify gene content.

## Background

Atlantic salmon are part of the Salmonidae family which comprise all salmon, trout, whitefish, grayling, and charr. A tremendous amount of basic biology is already known about salmonids from studies carried out on their physiology, population dynamics, behavioural ecology and phylogenetics [1]. Salmon also provide an excellent model system in which to study fundamental genetic mechanisms of growth, development, reproduction and response to infection and disease. For example, salmonids serve as prominent models for studies involving environmental toxicology [2], carcinogenesis [3], comparative immunology [4], the molecular genetics and physiology of the stress response [5], olfaction [6], vision [7], osmoregulation [8], growth [9] and gametogenesis [10].

Answers to fundamental scientific questions can also be gained from the study of salmonid genomes. The ancestor of all extant salmonids underwent a whole genome duplication and after a series of subsequent genetic events, salmon are now considered to be pseudo-tetraploid. How a genome reorganizes itself to cope with a duplicated genome and the importance of gene duplications for evolution and adaptation are long standing issues that remain unresolved. Questions regarding the origins of genomes have direct implication for our understanding of the roles of gene families, duplication and deletion of segments of genomes, and the mutational process in human health and disease. They also provide a foundation for understanding the genome of Atlantic salmon to benefit conservation and enhancement of wild stocks, aquaculture and environmental assessments. Genomic resources enable us to address fundamental scientific questions concerning the evolution of salmonid genomes, and the expression of genes and proteins in a wide variety of natural and altered environments and conditions.

Toward these goals, more than 175 cDNA libraries have been constructed from a wide variety of tissues and different developmental stages and more than 300,000 salmonid cDNA sequence reads have been combined from a consortium comprising groups from Canada (Ben Koop et al. and the Genomics Research on Atlantic Salmon Project (GRASP); Susan Douglas et al. and the Institute for Marine Biosciences, NRC); France (Yann Guiguen et al. and INRA-SCRIBE); Norway (Bjorn Hoyheim et al. and the Norwegian School of Veterinary Science (NSVS)) and the U.S.A. (Caird Rexroad III and the USDA/ARS National Center for Cool and Cold Water Aquaculture). These sequences were assembled into over 40,000 unique contigs. A preliminary microarray of 3,557 cDNAs was constructed and assessed on its' ability to provide new data in the study of cellular and tissue responses to pollutants, diseases and stress, as well as for reproduction and development [11-15]. On the basis of these results, a larger array of 16,006 genes has been constructed and initial results have shown sensitivity of gene expression patterns to disease challenge, and to small environmental and physiological changes [16].

## Results and discussion

Library construction (directional cloning by 5'*Eco*RI, 3'*Xho*I in pBluescript II XR, Stratagene; or TOPO TA cloning of suppression subtractive hybridization PCR products, Invitrogen and Clontech) and subsequent EST sequencing (using M13 forward primer) were designed to generate 3'-end sequences to enable us to distinguish between potential paralogs arising from the recent salmonid genome duplication. We have determined from a weighted average measurement comparing four different directionally-cloned library types (such as non-normalized versus normalized libraries) that approximately 9% of inserts are in the reverse orientation and therefore yield 5' sequence with the M13 forward primer [11]. The GRASP 3'-end reads were used as a framework on which to build the contigs from additional data provided by the NRC, INRA, USDA/ARS and the NSVS. Part of the evaluation process for selecting genes for the microarray required criteria that would guard against chimeras. Simply put, this meant that each gene choice had to be part of a contig with multiple distinct clones covering each region, or that it was sufficiently similar to another sequence across its whole length that it was unlikely to be chimeric. We did select for immune-specific and reproduction-relevant genes for the microarray, but the preponderance of ESTs on the 16K chip were randomly picked based on EST cluster quality and uniqueness and therefore represent a wide variety of different classes of genes.

### Application of a 16K cDNA microarray to different species

To explore the validity of using the 16K microarray with other fish species, the 13,421 Atlantic salmon (AS) and 2,576 rainbow trout (RT) cDNA features were interrogated with labeled liver targets from four members of the order Salmoniformes (AS, RT, chinook salmon and lake whitefish) and one member of the order Osmeriformes (rainbow smelt) (Table 1). The average percentage binding of AS, RT, chinook salmon, lake whitefish (LW) and rainbow smelt liver targets to the 16K chip was 54.0%, 63.3%, 51.0%, 50.6% and 30.1%, respectively. The average percentage of targets bound to AS and RT features for each species are also shown (Table 1).

**Table 1:** Determination of features bound by labeled cDNAs from different species on the 16K salmonid microarray.

	Average % bound to all features^a^	Average % bound to *S. salar *features^a^	Average % bound to *O. mykiss *features^a^
*S. salar *(n = 8)	54.0 ± 7.8	52.6 ± 7.9	59.5 ± 7.5
*O. mykiss *(n = 4)	63.3 ± 9.1	60.7 ± 9.6	74.4 ± 6.6
*O. tschawytscha *(n = 4)	51.0 ± 4.3	48.3 ± 4.4	62.9 ± 4.8
*C. clupeaformis *(n = 2)	50.6 ± 2.1	48.9 ± 2.4	57.8 ± 0.9
*O. mordax *(n = 2)	30.1 ± 3.5	28.8 ± 3.2	35.1 ± 5.4

^a^Average % bound ± standard deviation

Our study indicates that there are no significant differences in the percent of targets that bound to the 16K microarray for the four salmonids examined (AS, RT, chinook and LW). There is a similar hybridization performance for all salmonids. However, RT targets do consistently show higher overall binding to the microarray; the reason for this efficiency is not yet clear.

The hybridization performance of the rainbow smelt targets were roughly one-half those of the salmonid cDNAs. Of the species contributing targets to our heterologous hybridization experiment, the osmerid targets were the most phylogenetically removed from the salmonid features. Indeed, a recent mitogenomic study places the Osmeroidei in a separate clade from the Salmoniformes [17]. These two clades are separated by at least 200 MY with the Salmonidae having undergone at least one genome duplication event since their divergence [18,19]. Other factors such as genome gene content (ie., numbers of paralogs) and genome size are likely to be factors affecting the overall degree of hybridization [11].

### Application of a 16K cDNA microarray to different tissues

Different tissues and organs exhibit differences in transcriptome complexity, depending on their cellular heterogeneity and differentiated specializations. The mRNAs of a typical somatic cell are divided into three classes based on their sequence complexity and diversity [20]. The most prevalent class consists of only a few mRNA species that comprise the abundant transcripts present in a cell. Often these transcripts are dedicated to cellular functions common to all tissues, but they usually represent genes that specify an organs' unique function. The high complexity class of mRNAs includes thousands (perhaps millions) of different mRNA species, each represented by fewer than 15 copies per cell [20].

However, it should be noted that some subsets of genes that have been thought to be unique to one organ have been found to be expressed in others. This has been demonstrated for transcripts in the brain-gonad axis, and is probably not exclusive to these organs. For example, mammalian pheromone/odorant receptors and specific piscine hormones and receptors of the brain are also expressed in the gonad [12,21,22]. To date, the biological functions of these transcripts in the gonad have not been determined, raising intriguing questions regarding multiplicity of functions for complex transcripts, even in diploid vertebrates such as mammals.

To determine the differences in the transcriptome complexity of seven different AS tissues and organs, the 13,421 AS and 2,576 RT cDNA features were hybridized with labeled targets from midgut, brain, spleen, muscle, ovary, kidney and testis (Table 2). The average percentage binding of midgut, brain, spleen, muscle, ovary, kidney and testis targets to the 16K chip was 64.4%, 54.7%, 54.6%, 52.8%, 51.0%, 49.7% and 30.2%, respectively. In general, about 45% of the salmonid microarray features were not bound by targets from the various AS tissues and organs.

**Table 2:** Determination of features bound by labeled cDNAs from different tissues on the 16K salmonid microarray.

**Tissue Type**	**Average % bound**
Brain	54.7 ± 12.2
Kidney	49.7 ± 10.5
Midgut	64.4 ± 5.1
Spleen	54.6 ± 0.8
Testis	30.2
Ovary	51.0
Muscle	52.8 ± 9.7
Liver	54.0 ± 7.8

### Application of a 16K cDNA microarray to the same tissue from cohorts

To determine the amount of gene expression variability that exists between individuals of a single species, we compared the transcriptomes of livers from three fish with identical histories. We compared the average percent of variation (or scatter) in expression of liver transcripts between cohorts 1 and 2 (liverpairs 1/2), cohorts 1 and 3 (liverpairs 1/3) and cohorts 2 and 3 (liverpairs 2/3). Two separate experiments of six hybridizations each were conducted with each liverpairing having one dye-flip.

Examining each individual array in the intraspecies study showed that the overall mean scatter was 12.6% (Table 3). When the liverpair arrays and their respective dye-flips were combined and averaged, the overall mean scatter was reduced to 9.7%. This indicates that systematic unequal dye incorporation exists resulting in high scatter values. This dye bias has been well-documented by other researchers [23-25] and illustrates the importance of incorporating dye swap pairs when performing microarray hybridizations whenever possible. The overall mean scatter was further reduced to 5.2% when the analysis included technical dye swap replicates between respective liverpairs (Table 3). This demonstrates that increasing the number of technical replicates in a microarray experiment is an important factor to consider for reducing random scatter. It is encouraging that the overall scatter between individuals from the same broodstock was quite low. Thus technical and biological variability across arrays and individuals can be significantly reduced by the investigator if the appropriate experimental design is employed.

**Table 3:** Determination of variation in liver transcriptome expression between three cohorts.

Mean scatter for individual arrays	Mean scatter for dye swap pairs	Mean scatter for technical replicates
12.6%	9.7%	5.2%

### Application of a 16K cDNA microarray to analyze BAC contents

To assess the use of the 16K array as a screening tool to identify the genes present in a salmonid BAC, the 13,421 AS and 2,576 RT features were interrogated with nebulized and labeled fragments from a single BAC whose sequence has been determined (Table 4). Analysis of our initial BAC hybridizations revealed that a high proportion of transposon-like sequences and long and short interspersed nuclear elements were binding to the array. It is known that many different repeat elements derived from once-mobile transposable segments comprise large portions of the Atlantic salmon genome [26-29]. In an effort to improve the specificity of target binding to the microarray for BAC hybridization, we employed a Cot-1 DNA protocol to reduce the binding of these repetitive elements (Table 4). The addition of Cot-1 DNA increased the number of expected genes identified and the number of hits for the expected genes by displacing many of the repeat family and transposon associated elements.

**Table 4:** Analysis of gene content in BAC hybridizations.

BAC Name	Expected Gene Number^a^	Hybridized BAC (no Cot)	Hybridized BAC (with Cot)	Repetitive Elements (no Cot)^b^	Repetitive Elements (with Cot)^b^	Transposon Associated Sequences (no Cot)^c^	Transposon Associated Sequences (with Cot)^c^
92I04	8	5	8	5	4	9	7

^a^BAC 92I04 was previously characterized. The assembled BAC sequence was BLASTED against the microarray gene identification list to determine the expected gene number. Repetitive elements, transposon-associated sequences and unknown ESTs were not included in the total. Only the top 50 hits were examined.

^b^Number of identified microsatellite and repeat family elements.

^c^Number of identified transposon, transposase and reverse-transcriptase associated sequences.

Although Cot-1 DNA did improve the ability to identify genes for the BAC we examined, Cot-1 DNA alone is not enough to block the complications that arise from repetitive elements in whole genome hybridizations. In preliminary comparative genomic hybridization studies we have found that even with Cot-1 DNA included in the hybridizations, the repetitive DNA segments found in salmonid genomes interfere with the interpretation of the data. Most investigators are not interested in these repetitive segments, but rather in the genes that are interspersed between them. Moreover, we have found that often these repetitive elements lead to false positives. Using other methods, such as including repeat-element amplified products with Cot DNA, as well as higher stringency washes, might improve binding specificities. We are currently working on various strategies to maximize blocking of this repeat element 'noise'.

## Conclusion

We validate and demonstrate the usefulness of the 16K microarray over a wide range of teleosts, even for transcriptome targets distantly removed from salmonids phylogenetically. We show the potential of the use of the microarray in a variety of experimental settings through hybridization studies that examine the binding of targets derived from different organs and tissues. Intraspecific variation in transcriptome expression is evaluated and discussed. Finally, BAC hybridizations are demonstrated as a rapid and accurate means to identify gene content. We expect that this array will serve as an important resource for genetic, physiological, ecological and many other fields of salmonid study.

## Methods

### Gene selection

cDNA library construction, recombinant plasmid preparation and extraction, sequencing, sequence analysis and contig assembly for the GRASP have been described previously in detail [11-13]. Selection criteria for unique Atlantic salmon (AS) and rainbow trout (RT) cDNAs for inclusion on the 16K microarray were as follows: ESTs (cDNA fragments) were assembled into contiguous sequences (contigs) by PHRAP [30] under stringent assembly parameters (minimum overlap score:100; repeat stringency: 0.99). Contig consensus sequences and singleton sequences were aligned with non-redundant GenBank nucleotide and amino acid sequence databases using BLASTN and BLASTX, respectively [31,32]. Threshold for a significant BLAST hit was set at E = 1e-15.

It was determined that a contig must contain at least one "usable" sequence, where "usable" was a)- the sequence must be 3' (with high probability; containing polyA signal or having been sequenced with an oligo-dT primer or being at the 3'-end of a contig, with orientation determined by a strong hit against a protein in GenBank's non-redundant protein database), b)- be a sequence stretch containing more than 400 bp, and c)- the sequence must be at least 95% similar to the consensus of the contig.

It was also determined that if a contig was a singleton or singleton-equivalent (where all sequences were from the same plate or library thus not providing sufficient evidence for non-chimera status), then the contig selection was reinforced either by a)- a significant BLAST hit, E<1e-15 (BLASTN or BLASTX), or b)- it having 94% (or more) identity with a homolog (either paralog or ortholog) covering at least 400 nucleotides. If the contig was a non-singleton, it was determined that it must be a)- one "block" (having no regions in the interior of the contig covered by only one sequence, to decrease probability of chimeras), and b)- of high enough overall quality (with an overall score > 95% positions without conflicts, weighted by number of sequences which support the consensus) and c)- have few leading and trailing singleton positions (no more than 25%), since such positions make it a de facto singleton.

Approximately 3,500 additional sequences were selected with the following criteria: a)- no chosen contig could have 94% or more identity with another chosen contig, and b)- tentative consensus sequences (TC) identified by TIGR [33] could be included. By these criteria, approximately 1000 clones were picked indiscriminately from both normalized AS and RT cDNA libraries, 800 clones were selected from suppression subtracted hybridization libraries and 700 sequences were added from requests of potential array users. Additionally, 949 non-overlapping sequences (856 AS, 93 RT) from clones included in the preliminary 3,557-gene chip (plus one T cell receptor beta) were selected. Finally, approximately 500 immune-specific genes were also chosen to bring the total number of genes represented on the chip to 16,006. In the 16,006 cDNA features there are 13,421 AS, 2,576 RT, 4 chinook salmon, 3 rainbow smelt and 2 LW representatives.

### Gene identification

EST contigs were built using cDNAs on the array as reference and all ESTs currently in the GRASP database. Subsequent to microarray fabrication, the consensus sequences were screened for repeats using a custom salmonid repeat database with RepeatMasker. Masked consensus sequences were compared to GenBank databases. Using the stringent selection threshold above, the current percentage of the 16K features that are known and unknown genes is 55.8% and 44.2%, respectively. Analysis at less stringent thresholds is ongoing to identify all genes on the microarray.

### Microarray fabrication

Clones were robotically rearrayed from daughter glycerol stock 384-well plates into 96-well plates pre-filled with 7% glycerol in LB + ampicillin, incubated overnight at 37°C, and checked for uniform optical density. Plasmid inserts were PCR amplified in a Tetrad PTC-200 thermocycler (MJ Research) using 1 ul overnight culture, 0.2 mM M13/pUC forward primer (5'-CCCAGTCACGACGTTGTAAAACG-3'), 0.2 mM M13/pUC reverse primer (5'-AGCGGATAACAATTTCACACAGG-3'), 2 mM MgCl_2_, 10 mM Tris-HCl, 50 mM KCl, 250 mM dNTPs, 1U AmpliTaq (Perkin Elmer), and nuclease-free H_2_O (Gibco) to 100 ul. PCR conditions were: 2 min for 95°C; then 35 cycles of 95°C for 30 sec, 60°C for 45 sec, 72°C for 3 min; followed by 72°C for 7 min. Five ul of each PCR product were run on a 1% agarose gel to assess yield and quality. PCR products were robotically cleaned (Qiagen) and consolidated into 384-well plates, lyophilized by speed-Vac, and resuspended in 20 ul 3X SSC. Each purified PCR product concentration was determined and diluted to give a final concentration of 400 ng/uL.

All cDNAs were printed as single spots on EZ Rays aminosilane slides (Matrix/Apogent Discoveries) with the Biorobotics Microgrid II microarray printer (Genomic Solutions). Microspot™ 10K quill pins (Biorobotics) in a 48 pin tool were used to deposit approximately 0.5 nl (0.2 ng cDNA) per spot onto the slide. The slides were crosslinked in a UV Stratalinker 2400 (Stratagene) at 300 mJ. The resulting microarrays have a 4-by-12 metagrid layout with 19 X19 spot subgrid, each spot having an approximate diameter and pitch of 100 um and 0.20 mm, respectively. A 280 bp GFP (green fluorescent protein) cDNA was amplified from a GFP clone (Clontech) using the primers (5'-GAAACATTCTTGGACACAAATTGG-3') and (5'-GCAGCTGTTACAAACTCAAGAAGG-3') and printed in each subgrid corner to assist in gridding.

Six exogenous genome (Arabidopsis) cDNAs were amplified from the following clones kindly provided by The Arabidopsis Information Resource: rubisco activase [GenBank:T41667], protochlorophyllide reductase precursor [GenBank:R30630], chlorophyll a/b-binding protein CP29 [GenBank:N65746], PSII oxygen-evolving complex protein 2 [GenBank:H36167], tonoplast intrinsic protein root-specific RB7 [GenBank:AA067532] and ferredoxin (2Fe_2S) precursor [GenBank:W43249]. The Arabidopsis cDNAs were spotted in quadruplicate on each microarray and used for thresholding (determining number of transcripts present). Also, a ubiquitin normalizer serially diluted (50 pg, 5 pg, 500 fg, 50 fg, 5 fg, 0.05 fg and 0.005 fg) was applied to the array. Spot morphology was assessed by visual inspection, SYBR^® ^Green 1 (Molecular Probes) staining or hybridization with labeled non-specific probe. To check clone tracking, 47 high quality sequences were obtained from randomly-selected wells of the cleaned, consolidated 384-well plates used for microarray printing. Each tracked clone had BLAST identifiers matching gene IDs predicted from the re-array spreadsheet, indicating highly accurate clone tracking throughout the process of microarray fabrication.

### Animals

Various tissues (brain, kidney, midgut, spleen, ovary, testis, muscle) were sampled from two three-year-old AS (*S. salar*) adults (Pacific Biological Station, Nanaimo, B.C.). Livers were obtained from several 2.5 year-old AS (McConnell strain) and chinook salmon (*O. tshawytscha*) subadults (Fisheries and Oceans Canada, West Vancouver, B.C.). RT (*O. mykiss*) tissues (Spring Valley Strain) were obtained from Mountain Trout Sales (Sooke, B.C.). LW (*C. clupeaformis*) livers were obtained from three-year-old animals (Laboratoire Bernatchez, Université Laval, Quebec) and rainbow smelt (*Osmerus mordax*) livers were obtained from adult smelt (NRC Institute for Marine Biosciences and Memorial University of Newfoundland). Each institution that provided tissue, raised and treated the fish in compliance with ethics committee or government body guidelines.

### Tissue and RNA extraction

Fish were exsanguinated for several minutes. The tissues were removed and flash frozen in liquid nitrogen and stored at -80°C until RNA extraction. Flash frozen tissues were ground using baked (220°C, 5 h) mortars and pestles under liquid N_2_, then total RNA was extracted in TRIzol reagent (Invitrogen). RNAs obtained from these preparations were used for generating labeled targets for microarray hybridizations.

### Microarray hybridizations

The microarray experiments were designed to comply with MIAME guidelines [34]. To minimize technical variability, all targets were synthesized in one round and each hybridization experiment was conducted simultaneously on slides from a single batch where possible. Each hybridization experiment included dye-flips to compensate for cyanine fluor effects. Total RNA samples were quantified and quality-checked by spectrophotometer and agarose gel, respectively.

All hybridization experiments were performed using the SuperScript Indirect cDNA Labeling System kit and instructions (Invitrogen). Briefly, 5.0 ug total RNA was reverse transcribed using an anchored oligo d(T)_20 _primer in cDNA synthesis reactions that incorporated aminoallyl- and aminohexyl-modified nucleotides. The modified cDNAs were then labeled with fluorescent Cy5 or Cy3 dye in reactions with the amino-functional groups in coupling buffer.

### BAC DNA preparation

Previously sequenced Atlantic salmon BAC 92I04 obtained from the Children's Hospital Oakland Research Institute Atlantic Salmon BAC library (CHORI – 214) was isolated and purified. A total of 30 ug BAC DNA was added to shearing buffer containing 10 mM Tris-HCl pH 7.5, 1 mM EDTA and 20% glycerol. The DNA was sheared into fragments to a concentrated mass of 1500 bp by nebulization in an Invitrogen nebulizer (Cat# 45-0071) at 30 psi of N_2 _and concentrated by ethanol precipitation.

A total of 5 ug of nebulized BAC DNA was combined with 7.5 ug of pd(N)_6 _random hexamers (Amersham Biosciences), heated to 100°C for 5 minutes and then cooled on ice for 5 minutes. BAC fragment probes were then generated using Klenow Fragment DNA Polymerase (exo^-^) (New England Biolabs) in the presence of amino-modified nucleotides (Invitrogen) and labeled with fluorescent Cy3 dye in coupling buffer (see above). Before hybridizations, labeled BAC with Cot-1 salmon DNA was heated to 100°C for 15 minutes, placed on ice for 5 minutes, then warmed to 37°C; labeled BAC without Cot-1 salmon DNA was heated to 80°C for 10 minutes and then cooled to 65°C, before application of treated BAC to microarrays (see below).

### Microarray preparation

All microarrays were prepared for hybridization by washing 2 X 5 min in 0.1% SDS, washing 5 X 1 min in MilliQ H_2_O, immersing 3 min in 95°C MilliQ H_2_O, and drying by centrifugation (5 min 2000 rpm in 50 ml conical tube). All slides were prehybridized in 5 X SSC, 0.1% SDS, 0.5% BSA for 1.5 h at 49°C. Arrays were briefly washed 2 X 20 sec in MilliQ H_2_O, then dried by centrifugation. Labeled DNAs were hybridized to prewarmed microarrays in a formamide based buffer (25% formamide, 4X SSC, 0.5% SDS, 2X Denhardt's solution) 16 h at 49°C. The arrays were washed 1 X 10 min in 49°C (2X SSC, 0.1% SDS), and then 2 X 5 min in (2X SSC, 0.1% SDS), 2 X 5 min in 1X SSC and 2 X 5 min in 0.1X SSC at room temperature, then dried by centrifugation.

### Microarray analyses

Fluorescent images of hybridized arrays were acquired immediately at 10 um resolution using ScanArray Express (PerkinElmer). The Cy3 and Cy5 cyanine fluors were excited at 543 nm and 633 nm, respectively, at the same laser power (90%), with adjusted photomultiplier tube settings between slides to balance the Cy5 and Cy3 channels. Fluorescent intensity data was extracted from TIFF images using Imagene 5.5 software (Biodiscovery). Quality statistics were compiled in Excel from raw Imagene fluorescence intensity report files. Features were sorted (16,006 salmonid spots each representing different cDNAs; 24 Arabidopsis spots representing 6 different cDNAs) and median signal values and mean numbers of salmonid features passing threshold were determined for Cy3 and Cy5 data separately.

For cross-species and tissue-on-tissue experiments, the hybridization performance of labeled targets to salmonid features was assessed as a percentage of features bound from the numbers of AS and RT features passing a hybridization signal threshold, defined as two standard deviations above Arabidopsis signal mean. No transformations or normalizations were performed on these data. Only features deemed present by Imagene 5.6.1 (excluding marginal and absent values) were used for analyses. We also analyzed some of these data at two standard deviations above empty spot mean signal intensity and found that this was a less stringent method of thresholding (data not shown).

Intraspecific liver and BAC hybridization data analysis (background correction, Lowess normalization, and fold change gene list formation) was performed in GeneSpring 6.1 (Silicon Genetics). All scanned microarray TIFF images, extracted ImaGene grid files, the gene identification file and ImaGene quantified data files are available on-line as supplemental data [35]. The data is deposited in NCBI's GEO repository under PLATFORM GPL 2716 [36].

## Authors' contributions

KRVS: co-creator of cDNA libraries; participated in coordination of project; performed organ complexity and intraspecific hybridization experiments; drafted the manuscript.

MLR: constructed and participated in the characterization of high-complexity cDNA libraries, and participated in the development of selection criteria for genes included on the 16006-gene microarray.

GAC: development and optimization of hybridization protocols; performed various species and BAC labeling hybridization experiments; data analysis for manuscript.

GDB: implemented the sequence processing pipeline (from screening and trimming reads to BLAST-identifying assembled contigs); contributed to the set of criteria for establishing that a read was not a chimera, and assisted in other computational aspects of the project.

ARG: contributed to gene selection and identification protocols and general informatics.

CCN: participated in the design and manufacture of the microarray.

WSD and BFK conceived of the study and supervised its design and coordination.

All authors read and approved the final manuscript.
